# Parallel Adaptation to Spatially Distinct Distortions

**DOI:** 10.3389/fpsyg.2020.544867

**Published:** 2020-11-20

**Authors:** Yannick Sauer, Siegfried Wahl, Katharina Rifai

**Affiliations:** ^1^Institute for Ophtalmic Research, University of Tuebingen, Tuebingen, Germany; ^2^Carl Zeiss Vision International GmbH, Aalen, Germany

**Keywords:** visual adaptation, distortions, motion aftereffect, natural scenes, psychophysics, visual system

## Abstract

Optical distortions as a visual disturbance are inherent in many optical devices such as spectacles or virtual reality headsets. In such devices, distortions vary spatially across the visual field. In progressive addition lenses, for example, the left and right regions of the lens skew the peripheral parts of the wearers visual field in opposing directions. The human visual system adapts to homogeneous distortions and the respective aftereffects are transferred to non-retinotopic locations. This study investigates simultaneous adaptation to two opposing distortions at different retinotopic locations. Two oppositely skewed natural image sequences were presented to 10 subjects as adaptation stimuli at two distinct locations in the visual field. To do so, subjects were instructed to keep fixation on a target. Eye tracking was used for gaze control. Change of perceived motion direction was measured in a direction identification task. The point of subjective equality (PSE), that is, the angle at which a group of coherently moving dots was perceived as moving horizontal, was determined for both retinal locations. The shift of perceived motion direction was evaluated by comparing PSE before and after adaptation. A significant shift at both retinal locations in the direction of the skew distortion of the corresponding adaptation stimulus is demonstrated. Consequently, parallel adaptation to two opposing distortions in a retinotopic reference frame was confirmed by this study.

## 1. Introduction

Many optical devices induce spatial distortions of the visual field as a part of their optical aberrations. An example is the progressive addition lens (PAL) (Meister and Fisher, 2008). But also other optical devices like virtual-reality-headsets (Kuhl et al., 2009) cause geometric distortions, which alter different features of visual perception, such as size, motion, form, and distance of objects (Faubert, 2002; Lord et al., 2002; Habtegiorgis et al., 2018b). This interference with visual perception can have negative impacts on day-to-day life in the form of nausea and discomfort (Johnson et al., 2007), distance misjudgment (Kuhl et al., 2009), or tripping (Timmis et al., 2010). Additional severity is given by the fact that usually optically induced distortions are not homogeneous but vary across the visual field. For example, in progressive addition lenses, distortions are oppositely oriented in the left and right periphery (Meister and Fisher, 2008) or in virtual-reality-headsets they are radially varying (Kuhl et al., 2009).

The human visual system copes with changes in the perception by visual adaptation. Visual adaptation is the change of information processing as a response to alterations in visual input statistics (Clifford et al., 2007; Webster, 2015). Adaptation processes take place over many levels of the visual system continuously changing perception as a reaction to changes of all kinds of visual features from simple attributes such as orientation (Jin et al., 2005), contrast (Bao and Engel, 2012), or motion direction (Clifford, 2002; Knapen et al., 2009), which are processed in early levels of the visual system, to more complex attributes such as facial features (Leopold et al., 2005). Adaptation expresses in a normalization of visual information, which may be a benefit for the visual system in the form of efficiency of coding information (Clifford et al., 2007) or supporting constancy of perception (Foster, 2011).

Optical distortions change multiple visual features at the same time. Depending on the feature content of the distorted stimulus, adaptation occurs in multiple cortical areas. The visual system can adapt to changed orientation of objects, which could be caused by distortions. The perceived orientation of test stimuli changes after adaptation to tilted stimuli. This is known for adaptation with simple rotated geometric patterns (Gibson and Radner, 1937), as well as natural stimuli with a preferred orientation (Dekel and Sagi, 2015). Also motion statistics of a stimulus are altered by distortions. Adaptation can change perceived motion direction as well as speed of a test stimulus (Anstis et al., 1998). Furthermore, there are experiments proving interaction between visual perception of form and motion features (Mather et al., 2012). Adaptation to still images depicting motion can evoke motion aftereffects (Winawer et al., 2008). Vice versa motion signals can influence the perception of form (Uttal et al., 2000; Apthorp et al., 2011).

In progressive addition lenses skew distortions are the prominent type of distortion. After adaptation to skew distorted natural stimuli, the perceived level of image skew of a static pattern changes (Habtegiorgis et al., 2017) as well as perceived motion direction of a test stimulus (Habtegiorgis et al., 2019). The first study also showed that during fixation, the aftereffect is transferred to retinal locations without adaptation stimulation. Thus, adaptation to homogeneous skew distortions is, at least in parts, independent of the retinal location. This result is an indication for the involvement of higher cortical levels with larger receptive field sizes and processing mechanisms for complex form and motion features. It is not clear what kind of aftereffects will occur when different retinal locations are exposed simultaneously to adaptation stimuli with different distortions, similar to the situation of progressive lens wearers. Spatially localized aftereffects have been shown for several different adaptation processes: Low and also mid-level visual features like orientation (Blakemore and Campbell, 1969), spatial frequency (Ejima and Takahashi, 1984), perceived numerosity (Burr and Ross, 2008), or duration perception (Johnston et al., 2006) presumably have a small receptive field size, allowing localized aftereffects. With natural stimulus, content processed in higher visual areas and also the receptive field size increases leading to the transfer of aftereffects. Even for simple geometric shapes, transfer of distortion aftereffects has been shown, leading to a changed perception of elongation or curvature after brief presentation of elongated or curved shapes preceding the test stimulus (Suzuki and Cavanagh, 1998). Consequently, the presence of localized aftereffects in distortion adaptation with natural stimuli is not clear and requires investigation.

In this study, we want to examine the rivalry between global aftereffects, as they would be expected from transfer of adaptation, and local aftereffects, which would occur for local independent adaptation. To discriminate between the two cases, we designed a psychophysical experiment in which the two types of adaptation would lead to aftereffects with opposite directions. We use an adaptation stimulus for which the transfer of aftereffects has been shown and a second adaptation stimulus is added with the opposite skew direction. Local adaptation to the second stimulus should lead to a shift of perception in the direction opposite to the aftereffect from transferred adaptation. Usually distortions of optical devices are not homogeneous but vary across the visual field. To cope with the changed visual input by adaptation, the visual system needs to be able to adapt locally with a spatial variation of aftereffect directions.

In this experiment, parallel adaptation to two distorted stimuli at spatially distinct locations is studied. The homogeneously but oppositely skewed adaptation stimuli in the form of natural image sequences are shown simultaneously at two distinct locations in the visual field with the same eccentricity. For the same skew distorted natural stimulus, both motion and form aftereffects are known to occur (Habtegiorgis et al., 2017, 2019), since both motion and form features are altered by skew distortions. Additionally, because of interaction between the processes of motion and form adaptation only one type of aftereffect is used in our experiment as measurement for skew distortion adaptation. Aftereffects were measured in a motion direction identification task, at the same retinal locations as the presentation of adaptation stimuli. Results show a simultaneous shift of perceived direction in opposing directions for both retinal test locations after parallel adaptation. Each shift corresponds to the skew direction of the corresponding adaptation stimulus.

## 2. Methods

Adaptation to spatially varying distortions was studied by presenting oppositely skewed natural image sequences at two distinct locations in the periphery simultaneously. Gaze was fixed centrally between the stimulus locations. Aftereffects to adaptation were measured in a motion direction identification task at the same two distinct locations.

### 2.1. Study Approval

The study was approved by the Ethics Committee of the Medical Faculty of the Eberhard Karls Universität Tübingen and the University Hospital.

### 2.2. Observers

Nine observers (six male and three female aged between 21 and 28) with normal or corrected-to-normal vision participated in the study. All but one were naive about the purpose of the study. The experiment was conducted in accordance with the Declaration of Helsinki and participants gave their informed written consent.

### 2.3. Set-Up

Stimuli were shown on a ViewPixx monitor (VPixx Technologies Inc., Canada) with a resolution of 1, 920 × 1, 200 pixels and a refresh rate of 120 Hz viewed at a distance of 65 cm. The monitor covered a visual angle of 41° horizontally and 24° vertically. Gaze of the subjects was controlled by an EyeLink 1000 Plus eye tracker (SR Research, Canada) at a sampling rate of 1 kHz. Viewing distance and head position was fixed by using a chin and forehead rest. The up and down keys on a keyboard were used by the subjects to respond in the test phase. The experiment was run with the Psychophysics Toolbox (Brainard, 1997) in Matlab (Mathworks, USA). Viewing was monocular.

### 2.4. Stimuli

#### 2.4.1. Adaptation Stimulus

**Figure 1:**
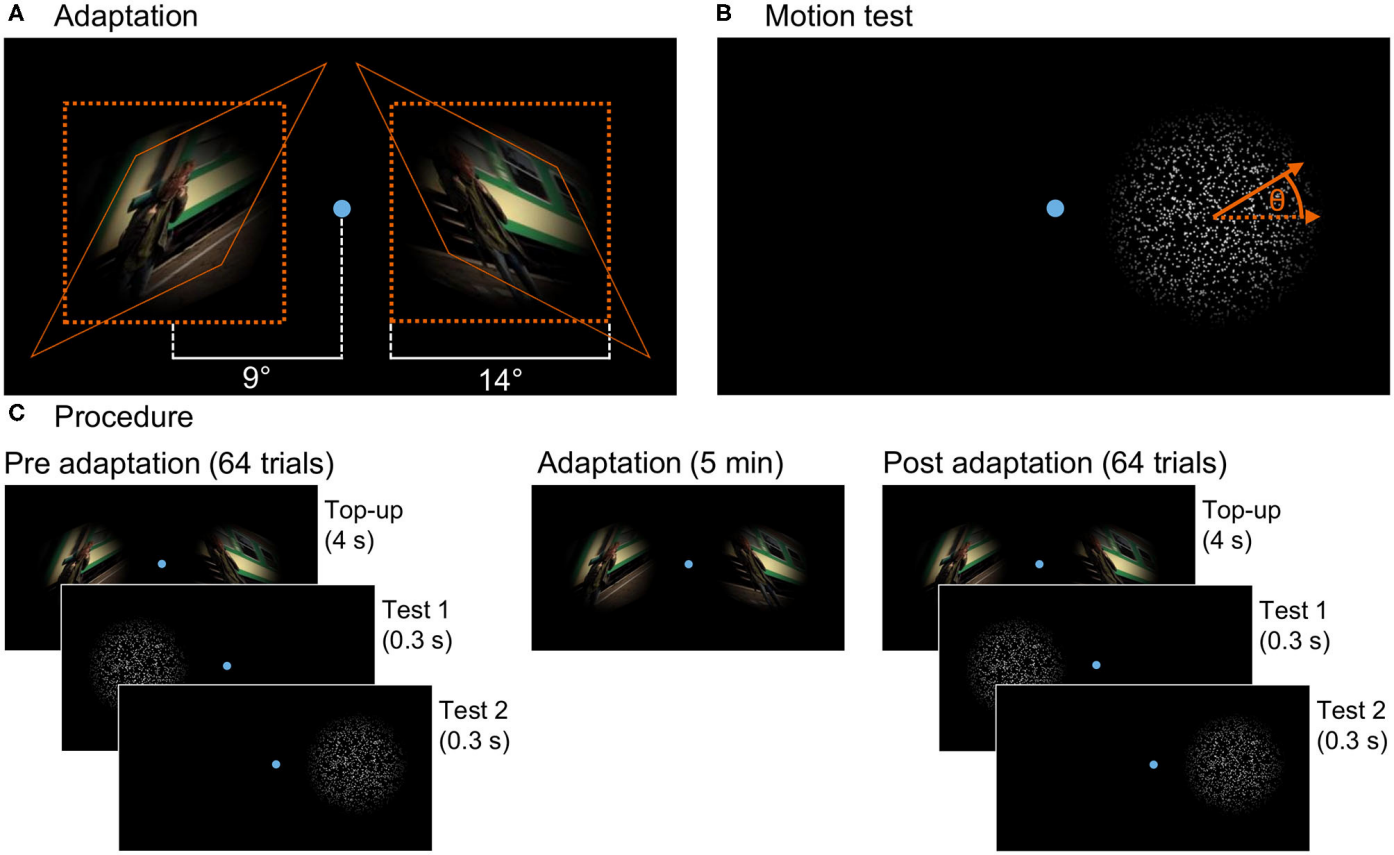
**(A)** Presentation in the adaptation phase: The figure shows one frame of both skewed stimuli and their placement on the screen together with the fixation point. The natural image sequence was skewed with angle θ = 25° for the left and θ = −25° for the right stimulus. After skewing, the stimuli were masked with a Hanning window function. The orange lines indicate how a square of the original video gets transformed by the skew mapping. **(B)** Test stimulus: Randomly distributed dots moving coherently in one direction with the angle θ to the horizontal randomly chosen out of a limited set for each trial. Here, the test for the right stimulus location is shown. During the experiment, both stimulus locations were tested sequentially. **(C)** Three phases of the experiment: In the pre- and post-adaptation phases, the motion direction perception test is presented at both locations on the screen (in a random order) interrupted by top-up adaptation stimuli in 64 trials each. In the adaptation phase, only the adaptation stimuli are shown for a duration of 5 minutes.

Adapting stimuli were generated by skew distorting natural image sequences from an open source movie (Baumann, 2010). The frames of size 1, 200 × 720 pixels were skewed using MATLAB (Mathworks, USA) by mapping the pixel position (*x, y*) in the undistorted frame to the new positions (*x*_*s*_, *y*_*s*_) given by the transformation:

(1) $$\begin{array}{l} \left(\begin{array}{c} x_{s}\\ y_{s} \end{array}\right)=\left(\begin{array}{c} x+y\tan\theta \\ y+x\tan\theta \end{array}\right) \end{array}$$

with the angle θ defining a shear mapping in vertical and horizontal direction with the same amount. To reduce boundary effects, the frames were cropped to a size of 650 × 650 pixels and masked with a Hanning window function

(2) $$\begin{array}{l} w\left(r\right)=\cos^{2}\left(\frac{\pi r}{N}\right) \end{array}$$

where *r* is the distance to the center of the frame and *N* = 650 pixels is the width of the frame. Two kinds of stimuli, with opposite skew of θ = 25° and θ = −25°, were prepared. They are shown in Figure 1A.

#### 2.4.2. Test Stimulus

To test adaptation aftereffects, a random dot test was used. White dots with diameter 5 pixels on a black background were randomly positioned within a circle of diameter 14° visual angle and moved coherently at a speed of 6.6° visual angle per second. Direction of motion was diagonally upwards or downwards randomly to the left or right with an angle to the horizontal chosen randomly out of ±8, ±6.5, ±5, ±3.5, ±2, ±0.5°. Dots moving outside of the circle were randomly repositioned at the opposite side of the circle (φ∈[90°+θ, 270°+θ]). The dot stimulus was masked with the same Hanning window function as the adaptation image sequences.

### 2.5. Procedure

Subjects were lead into the study room, seated on a chair, and introduced to the experiment procedure. The room was darkened and subjects performed a few test trials before the actual experiment started. Three different phases of the experiment are illustrated in Figure 1C. In the pre-adaptation phase, the baseline perception of motion direction was assessed. In repeated trials, random dots moved on the screen and subjects answered the perceived direction of motion. The second phase was the adaptation phase. Two oppositely distorted adaptation stimuli were shown simultaneously on the screen. The perception after adaptation was measured in the post-adaptation phase in a procedure similar to the first phase.

As illustrated in Figure 1, two distinct stimulus locations on the screen were used for both adaptation and the test stimuli. They were on the left and right side of the screen with their centers both at a distance of 9° visual angle to the screen center. In the middle between the stimulus locations during the whole experiment, a small dot (14 pixels in diameter, 0.3° visual angle) indicated the fixation target for the subjects.

In the trials of the pre-phase, the moving dot test was performed consecutively on the left and right side with the first position chosen randomly from the two stimulus locations. The dots moved for 0.3s randomly to the left or right with an angle to the horizontal chosen randomly form the given set, after which subjects answered the perceived direction of motion by clicking up or down on a keyboard. The experiment continued only after a valid key was pressed. Then, after a 0.5-s break after the key press, the test was repeated at the opposite stimulus location. Each of these trials was followed by a short top-up adaptation of 4 s. In the pre-phase, undistorted image sequences were used and presented simultaneously at both stimulus locations. After a break of 0.5 s, the next trial started with a random dot test. In 64 trials, each stimulus angle was tested 6 times (only 4 times for large angles of ±8 and ±6.5°) in a randomized order.

The second phase induced adaptation by presenting the skewed images sequences for 5 min without interruption. Always oppositely distorted stimuli were used for the two stimulus locations, but location was randomly interchanged between subjects. Also in this phase the fixation point was shown in the center of the screen.

The third phase, the post-adaptation phase, was similar to the first phase, but distorted stimuli consistent with the adaptation phase were used as top-up adaptation.

To ensure that always the same retinal locations were stimulated, during adaptation and test the subjects' gaze was controlled and the adaptation stimuli vanished in <20 ms (under two monitor refresh cycles Saunders and Woods, 2014) when subjects blinked or their gaze deviated from the fixation point by more than 2°. The stimuli appeared back as soon as measured gaze was inside the fixation area again. In the test trials, the subjects' gaze was controlled before presentation of the stimulus and the dots were only shown when gaze was inside the fixation area. Otherwise, the current trial was aborted and repeated at the end of the phase.

## 3. Analysis

To measure aftereffects following adaptation to skewed natural image sequences, the change of perceived motion direction was evaluated by comparing the stimulus level perceived as a horizontal movement between pre- and post-adaptation phases.

The percentage of trials answered upwards depending on the stimulus level was determined for both stimulus locations in the pre- and post-phase. Dots perceived as moving to the left diagonally downwards correspond to the same axis of motion (and therefore the same level of skew distortion) as dots moving to the right but upwards. Thus, to collapse data from trials with different horizontal motion direction for analysis, answers for trials with movement to the left were inverted. The obtained curves of percentage of upwards answers depending on stimulus level were fitted for every subject with a psychometric function (cumulative normal distribution function with free but equal asymptotes) using Psignifit (Schütt et al., 2016) in Matlab. The 50% point of the fit function is used as a measurement for the point of subjective equality (PSE), that is, the stimulus level in degree which is perceived as a horizontal motion. The shift ΔPSE = PSE_post_−PSE_pre_ between pre- and post-adaptation phases is computed as a measurement of aftereffects.

To collapse data from subjects with positive skew on the left and negative on the right stimulus location and other subjects with negative skew on the left and positive skew on the right stimulus location, in our analysis all subjects were treated as having positive skew at the left location and negative skew at the right location by inverting ΔPSE for subjects with the negatively skewed adaptation stimulus at the left stimulus location. In this way, for the left stimulus location a positive shift ΔPSE represents a change of perceived motion direction (into the negative) opposing the skew direction of the corresponding adaptation stimulus. For the right stimulus location, ΔPSE is negative if motion perception changes opposing the adaptation stimulus.

For statistical analysis, one-sample *t*-test was used with ΔPSE for the left and right stimulus location to test the presence of a shift in perceived motion direction. A paired *t*-test was conducted with both ΔPSE to evaluate the significance of the difference between both stimulus locations.

## 4. Results

**Figure 2:**
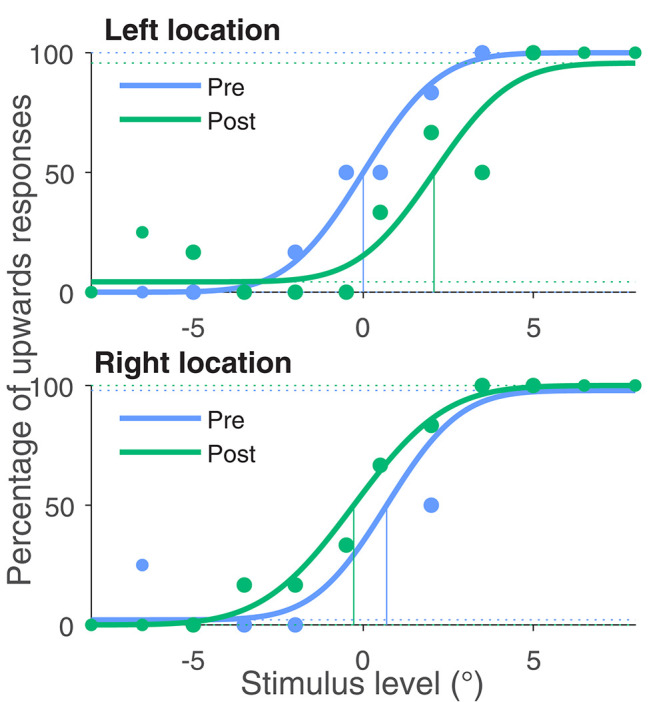
Percentage of upwards responses and psychometric fits for one exemplary subject. The blue data points were obtained in the baseline measurement in phase one of the experiment and the green data points in the last phase after adaptation. All four curves were fitted with a psychometric function. The 50% point of the fit function is taken as the Point of Subjective Equality (PSE). The PSE for this subject shifted by 2.1° for the left stimulus location, where the adaptation stimulus was left skewed (negative skewing angle). For the right stimulus location, this subject's PSE shifted by −1.0° in accordance with the positive skewing angle.

Figure 2 shows the number of upwards answers depending on the stimulus level and the fits of the psychometric function for one representative example subject. At the left stimulus location, where the adaptation stimulus was skewed with an positive angle, the PSE shifted by 2.1°. At the second location at the right side of the screen, there is a negative shift ΔPSE = −1.0°. This means that for the left side motion, direction is perceived by this subject more downwards and on the right side more upwards. So motion direction perception has shifted away from the adaptation stimulus at both locations.

This change of perception indicates that the subject saw both image sequences less distorted after adaptation than in the beginning of adaptation.

**Figure 3:**
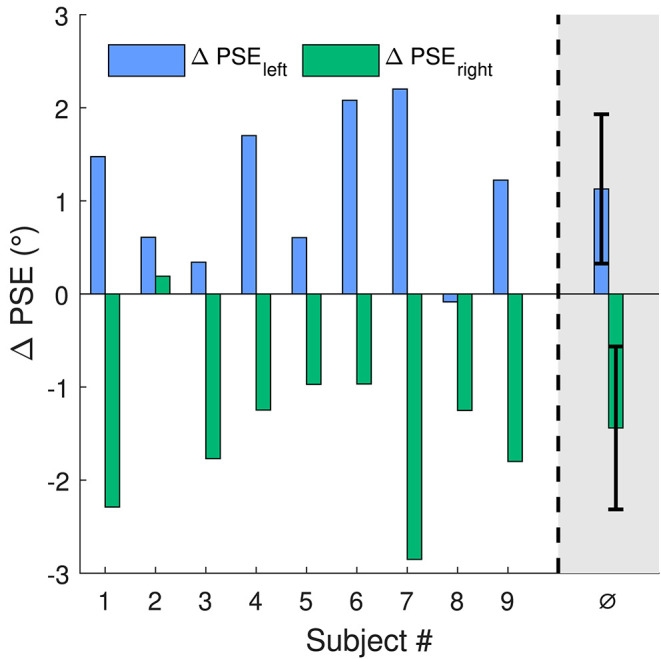
Shifts of PSE for the 2 stimulus locations on the screen. For location one (blue bars), to agree with the right skewed adaptation stimulus, a positive shift of PSE is expected. For the second location (green bars) with the oppositely skewed adaptation stimulus, the expected shift in PSE is negative. The rightmost bars are the mean values of ΔPSE with standard deviation as error bars.

Figure 3 shows ΔPSE for all subjects at both stimulus locations. On the left side, all subjects but one show a positive shift PSE (*p* < 0.01 for ΔPSE_left_). On the right side, for all subjects but one the shift of PSE was negative (*p* < 0.01 for ΔPSE_right_). Thus, the shifts of perception were always in opposite directions for the left and right side (*p* < 0.001 for ΔPSE_left_−ΔPSE_right_). In average, the shift for the left stimulus position was 1.13° (s = 0.8°) and for the right position −1.4° (s = 0.9°). Skew direction of the adaptation stimulus was positive for the left and negative for the right stimulus location. This means the change of motion direction perception was opposing to the skew direction of the corresponding adaptation stimulus at both stimulus locations.

## 5. Discussion

This study assessed the parallel presence of spatially separated aftereffects after adaptation to two spatially distinct opposing distortions in a psychophysical experiment. Subjects were exposed to two homogeneously but oppositely skew distorted natural image sequences shown at two distinct locations in the visual field at identical eccentricity. Aftereffects were measured at the identical locations by evaluating the shift of perceived motion direction measured in a direction identification task. The results show opposing shifts of PSE at the left and right side. At both stimulus locations, the direction of aftereffects opposes the skew direction of the corresponding adaptation stimulus. This shows that the human visual system is able to adapt to multiple, spatially separated distortions simultaneously. Perception is changed locally in the visual field depending on the distortions present at the test locations. So distortion adaptation does not only take place globally, but can vary across the visual field.

Aftereffects result from response changes of neurons tuned to attributes changed by the adaptation stimulus (Webster, 2015). Adaptation to features processed in lower levels, like tilt (Mathôt and Theeuwes, 2013) or contrast (Gardner et al., 2005), shows purely retinotopic aftereffects, meaning aftereffects are present only at locations exposed to the adapting stimulus but not transferred to non-adapted locations. Neurons in higher cortical areas have larger receptive field sizes (Van Essen and Anderson, 1995; Suzuki et al., 2005), therefore aftereffects are at least partially transferred to non-adapted retinal locations, like it has been shown for adaptation to complex facial features (Zimmer and Kovacs, 2011). For this face aftereffect (FAE), it has also been shown that the effect size decreases in case of simultaneous presentation of conflicting stimuli (Afraz and Cavanagh, 2008). For distortion adaptation with natural stimuli also higher cortical levels are involved, since it has been shown that distortion adaptation aftereffects are transferred to non-adapted locations (Habtegiorgis et al., 2017). The presence of multiple opposing aftereffects in our experiment again suggests involvement of lower areas, where different neuron populations are able to adapt differently for different retinal locations. The question arises whether adaptation is aggravated in case of simultaneous adaptation with two opposing stimuli. This could be seen in a smaller effect size when comparing aftereffects in our experiment to a condition with only one adaptation stimulus. If we take the change in angle of perceived motion direction as a direct measurement for perceived skew distortion, meaning the skew angle θ corresponds to ΔPSE, the effect size in our experiment is comparable to the previous study showing global adaptation aftereffects (Habtegiorgis et al., 2017).

The motion direction aftereffect is known to be retinotopic, also with locally opposing aftereffects (Wenderoth and Wiese, 2008). So the results of our experiment could be explained by adaptation to changed motion direction alone. But the natural image sequence used in this experiment as adaptation stimulus contains a variety of motion as well as form features. It is well-known that form adaptation influences motion perception and vice versa (Winawer et al., 2008; Mather et al., 2012; Pavan et al., 2013). So the change of orientation content by distorting the adapting stimulus can induce motion direction aftereffects. At the same time, the altered motion direction statistics of the natural image sequences can influence the perceived skew after adaptation. Both types of aftereffects, change of perceived skew as well as motion direction, are known to occur for skew distorted natural stimuli (Habtegiorgis et al., 2017, 2019). This all suggests that the tested motion aftereffect is a measurement for distortion adaptation. The results show that skew adaptation can take place simultaneously for two stimuli with opposing skew directions and not only with a global homogeneous aftereffect.

Another possible explanation of our results is adaptation to curvature of a three-dimensional surface: Two distorted adaptation stimuli combined could stimulate detectors for curvature of a surface covering the area of both stimulus locations (Suzuki, 2001). Opposite skew distortions, as used in this study, fit to a parallel projection of the undistorted stimulus on two oppositely inclined planes. In this geometrical configuration, the stimuli would then lie on different sides of a three-dimensional object, with the intersection line of the two inclined planes between them. Adaptation to the curvature of the surface would then also lead to a shift of perceived motion direction in opposite directions. There are some reasons why the stimulation of a corresponding surface curvature detector is not guaranteed in our setup: Image skew is ambiguous in the tilt of the three-dimensional-oriented planar surface, the undistorted image is projected on, similar to an ellipse which has two interpretations in 3D (Stevens, 1983). Therefore, a concave as well as convex configuration of planes could distort the two stimuli in the same way. Furthermore, our stimulus setup does not show a continues curvature, meaning the point between the two stimulus locations where the hypothetical surface inclination would change is in fact not part of given visual information. It has been shown that for curvature adaptation such a gap in the adapting stimulus drastically reduced aftereffects (Gheorghiu et al., 2009). For the perception of a surface curvature form motion, there needs to be a change in the second derivative of the optic flow field (Droulez and Cornilleau-Pérès, 1990). But this is not introduced by skew distortions, as it is a linear transformation of spatial coordinates. The orientation of the hypothetical rotated planes could be perceived by motion, but again there is an ambiguity of the plane tilt because of the linearity of the skew transformation (Zhong et al., 2006). Combined, it is not clear which role curvature adaptation plays in the process of adaptation to opposite skew distortions and requires further investigation.

The process revealed by this study is an important part of understanding of how the visual system copes with optically induced distortions. The results show that humans' visual system can reduce the amount of perceived spatially varying distortions by adaptation. Wearers of PALs or VR-glasses benefit from this process by a decrease of problems like discomfort and nausea. In the case of optically induced distortions, additional complexity arises by the fact that varying distortions are in general not spatially distinct and homogeneous but gradually change across the visual field. Also the distortions in the visual field are constantly modulated by eye movements. The distortions are not fixed relative to the retina, as it is the case in this study, but mostly relative to the head and therefore change upon gaze. To reduce the perceived distortions in VR headsets or PALs, aftereffects would thus have to occur not relative to the retinal but the spatial coordinates of an adaptation stimulus. For a single homogeneously distorted adaptation stimulus, the transsaccadic transfer of aftereffects has been shown in retinotopic as well as spatiotopic reference frames (Habtegiorgis et al., 2018a). The spatiotopic adaptation process, in contrast to purely retinotopic adaptation, requires necessarily involvement of high level neurons (Duhamel et al., 1992; Nakamura and Colby, 2002; d'Avossa et al., 2007). Future studies might reveal the presence of this spatiotopic aftereffects after eye movements also for multiple opposing distortions.

## Data Availability Statement

The raw data supporting the conclusions of this article will be made available by the authors, without undue reservation.

## Ethics Statement

The studies involving human participants were reviewed and approved by Ethics Committee of the Medical Faculty of the Eberhard Karls Universität Tübingen and the University Hospital. The patients/participants provided their written informed consent to participate in this study.

## Author Contributions

All authors developed the study idea and procedure. YS performed the experiment and the analysis. All authors discussed the results and contributed to the final manuscript.

## Conflict of Interest

SW and KR are employees of Carl Zeiss Vision International GmbH, as detailed in the affiliations. The remaining author declares that the research was conducted in the absence of any commercial or financial relationships that could be construed as a potential conflict of interest.
